# Information on 'Overdiagnosis' in Breast Cancer Screening on Prominent United Kingdom- and Australia-Oriented Health Websites

**DOI:** 10.1371/journal.pone.0152279

**Published:** 2016-03-24

**Authors:** Alex Ghanouni, Susanne F. Meisel, Jolyn Hersch, Jo Waller, Jane Wardle, Cristina Renzi

**Affiliations:** 1 Health Behaviour Research Centre, Department of Epidemiology and Public Health, University College London, 1–19 Torrington Place, London, WC1E 6BT, United Kingdom; 2 School of Public Health, University of Sydney, Edward Ford Building, Sydney, NSW 2006, Australia; University of Torino, ITALY

## Abstract

**Objectives:**

Health-related websites are an important source of information for the public. Increasing public awareness of overdiagnosis and ductal carcinoma in situ (DCIS) in breast cancer screening may facilitate more informed decision-making. This study assessed the extent to which such information was included on prominent health websites oriented towards the general public, and evaluated how it was explained.

**Design:**

Cross-sectional study.

**Setting:**

Websites identified through Google searches in England (United Kingdom) and New South Wales (Australia) for “breast cancer screening” and further websites included based on our prior knowledge of relevant organisations.

**Main Outcomes:**

Content analysis was used to determine whether information on overdiagnosis or DCIS existed on each site, how the concepts were described, and what statistics were used to quantify overdiagnosis.

**Results:**

After exclusions, ten UK websites and eight Australian websites were considered relevant and evaluated. They originated from charities, health service providers, government agencies, and an independent health organisation. Most contained some information on overdiagnosis (and/or DCIS). Descriptive information was similar across websites. Among UK websites, statistical information was often based on estimates from the Independent UK Panel on Breast Cancer Screening; the most commonly provided statistic was the ratio of breast cancer deaths prevented to overdiagnosed cases (1:3). A range of other statistics was included, such as the yearly number of overdiagnosed cases and the proportion of women screened who would be overdiagnosed. Information on DCIS and statistical information was less common on the Australian websites.

**Conclusions:**

Online information about overdiagnosis has become more widely available in 2015–16 compared with the limited accessibility indicated by older research. However, there may be scope to offer more information on DCIS and overdiagnosis statistics on Australian websites. Moreover, the variability in how estimates are presented across UK websites may be confusing for the general public.

## Introduction

Overdiagnosis can be defined as the detection of a disease that would not cause symptoms or death if it had remained undetected. It is a widely debated issue in breast cancer screening since it can lead to unnecessary treatment (i.e. ‘overtreatment’), including surgery, radiotherapy, hormone therapy, and chemotherapy. These can be associated with both short- and long-term adverse effects in addition to the negative psychological consequences of becoming a breast cancer patient [1]. Much overtreatment in breast screening is suspected to arise from the unnecessary and unbeneficial detection of ductal carcinoma in situ (DCIS). Although DCIS can become invasive, symptomatic, and a threat to life, it can also remain confined to the milk ducts in which it arose, remaining asymptomatic throughout a woman’s lifetime [2].

Previous research has found both limited public awareness of overdiagnosis and widespread misconceptions about it. When a survey sample of 500 Australian adults were asked what they thought the term meant, only 41% were able to provide a definition that was considered approximately correct (e.g. *“exaggerating something that’s there”*) [3] and a similar United Kingdom (UK) study of 390 adults found that less than 1% of the general public could provide a more precise definition (e.g. *“diagnosis of a disease that will not cause symptoms or death”* (Ghanouni et al., under review). Members of the public often have difficulty understanding the concept, and find it counterintuitive when it is explained [4, 5]. This suggests that there is substantial scope to improve how information on overdiagnosis is communicated.

A 2012 US survey found that 54% of adults aged 50 to 64 years had used the internet to search for health information in the past year [6], suggesting that health websites are likely to be an important way in which health concepts and recommendations are disseminated. In the UK, the Cancer Research UK website [7] alone regularly receives over 1 million visits per month [8]. However, a previous study evaluating 27 websites on breast cancer screening concluded that information on overdiagnosis (and overtreatment) was often omitted or inaccurate [9]. To our knowledge, although this study was published in 2004 (websites themselves were accessed in 2002), it is the most recent appraisal of online information relating to overdiagnosis in breast screening. However, there have been notable developments since then. For example, controversy around whether screening invitees were being adequately informed about overdiagnosis [10] resulted in the commissioning of the Independent UK Panel on Breast Cancer Screening review (IUKPBCS) [2, 11] to estimate the magnitude of overdiagnosis and breast cancer mortality reduction attributable to screening. Although health websites might be expected to have incorporated the review’s findings into their information materials, this has not been formally evaluated. Moreover, the web in 2016 bears little resemblance to the web as it existed in 2002. Availability of broadband and internet usage among the general public have increased [12], creating a greater incentive for producers of health content to provide high-quality web-based information [13]. In addition, the increased bandwidth available to home users has allowed content producers to offer information in formats other than simple text and images, such as streaming video.

We evaluated information on overdiagnosis and DCIS provided by health websites oriented towards the general public in the UK and Australia (and England and New South Wales). These two countries were selected to allow an international analysis of English-language websites in areas where breast cancer screening is offered as part of organised screening programmes that is free at the point of use. We looked at whether any such information was provided, its content, and how it compared across websites, with particular focus on quantitative information describing the magnitude of overdiagnosis in breast screening.

## Materials and Methods

### Website identification

Google has approximately a 90% market share among search engines [14], making it an important intermediary between individuals seeking information on breast cancer screening and the information source itself. Consequently, websites were selected based on a pair of Google searches for “breast cancer screening”, carried out on 13^th^ August 2015 for UK-oriented websites and 14^th^ December 2015 for Australia-oriented websites. These were performed using an ‘incognito’ window in Chrome (for Windows; Google, Mountain View, CA, USA) in order to prevent cookies and user account data from influencing search results. The two searches were carried out in the respective countries of interest, in order to ensure that results were tailored to users from those countries. The first five pages of results (using the default number of results per page) were scanned manually in order to identify potentially relevant websites. This was anticipated to identify the vast majority of websites that users might realistically access: “click-through rates” for links are less than 5% for websites listed lower than the top ten results [15].

The Google search was supplemented with the authors’ prior knowledge of resources that contained relevant information and were anticipated to be commonly used. Search results were excluded based on the following criteria: i) duplicate information sources (e.g. two links to the same website), suggested secondary search queries (e.g. “breast cancer screening age”), or subsidiary links (e.g. search results linking to information pages on “diagnosis” and “treatment” within a single main result); ii) research articles, technical reports, or sites aimed at healthcare professionals; iii) media releases or media articles (e.g. news stories); iv) websites from organisations with a remit outside the UK or Australia, as applicable (e.g. the National Institutes of Health in the United States); v) websites with a remit specific to a country (or state/territory) within the United Kingdom (or Australia) other than England (or New South Wales; NSW); vi) locally- or regionally-oriented websites (e.g. those of city-specific or county-wide breast screening services). Although the web is generally accessible across national borders, healthcare and screening systems are organised at a national level within the UK and at a state level in Australia. Hence, we limited our analysis to websites from organisations that were either focused on breast screening in England or NSW, or that related to the UK or Australia as a whole, in order to obtain suitably homogenous sets of resources that could be interpreted within similar policy contexts. Finally, we excluded websites that did not provide information on breast cancer screening.

### Analysis

Websites were categorised as originating from either charities, health service providers, government agencies, or other groups. They were evaluated using content analysis [16] to determine i) whether they included any information on overdiagnosis, overtreatment or DCIS in breast screening, ii) how overdiagnosis or DCIS was described, and iii) how overdiagnosis was quantified. Three authors browsed through websites manually with the aim of identifying all primary information pages that referred to any of these aspects. Browsing was supplemented by using the search functions built into websites, where these were available. Where relevant information was located, the text was extracted and saved in an Excel 2010 spreadsheet (for Windows; Microsoft, Redmond, WA, USA) for subsequent interpretation and comparison. Descriptions of overdiagnosis, DCIS, and relevant statistics were evaluated independently by pairs of authors who read the extracted text to identify what they considered to be the key information points, which were then listed and summarised for each website. These were then counted and (where possible) combined, in order to generate the number of websites containing each information point. Authors compared their categorisations in case of any discrepancies, which were resolved through discussion. The content of information was also compared across websites to highlight similarities and differences.

Since all data were publically available and the study did not involve human participants, the host institution considered this study to be exempt from requiring ethical approval.

## Results

### Website selection

The initial Google searches yielded 167 and 105 links for the UK and Australia, respectively. Duplicate results, suggested secondary searches, and secondary subsidiary links were excluded (UK: n = 112; Australia: n = 57), leaving 55 and 48 unique search results.

UK websites: Nine websites consisting of research articles, technical reports, or resources aimed at healthcare professionals were excluded, as were 14 media articles, six websites with a remit outside the UK, five websites with a remit specific to a UK country other than England, and 11 regionally-focused websites within England. The ten remaining websites were searched for information relating to breast cancer screening and a further three were excluded for not providing any such information. Three websites were added based on authors’ prior knowledge for a total of ten websites to be included in the analysis.

Australian websites: Five research articles/professional resources and nine media articles were excluded. Nine websites with a remit outside Australia, nine region-specific websites outside NSW, and three region-specific websites within NSW were also excluded. Of the remaining websites, five were excluded for not including information about breast screening, leaving a total of eight websites to be analysed.

### Website characteristics

UK websites: Six websites were from health charities (Against Breast Cancer, Breast Cancer Care, Breast Cancer Now, Cancer Research UK, healthtalk.org, Macmillan Cancer Support) [7, 17–21], two were from health service providers (National Health Services [NHS] Choices, and Public Health England) [22, 23], and one was from an independent health website (Patient) [24]. In England, invitees to the NHS Breast Screening Programme are routinely posted an invitation leaflet that aims to provide enough information to allow women to make an ‘informed choice’ about their participation. An electronic version of the leaflet is accessible to the general public online (currently available via Public Health England but hosted on a separate website at the time of the analysis). Consequently, we evaluated this as well [25].

Australian websites: Three websites were from cancer charities (Breast Cancer Network Australia, Cancer Council Australia and Cancer Council NSW) [26–28], two were from breast screening service providers (BreastScreen Australia and BreastScreen NSW) [29–30], and three were from government agencies (Australian Institute of Health and Welfare, Cancer Australia, and Cancer Institute NSW) [31–33]. There was some overlap between organisations: the Cancer Council NSW is a member of the Cancer Council Australia (the national body of the charity). BreastScreen NSW is part of BreastScreen Australia (the national breast cancer screening programme) and is managed by the Cancer Institute NSW. As with the English Breast Screening Programme, the standard information leaflet provided to invitees in NSW is freely available online. It is hosted on the BreastScreen NSW website and so we considered it part of that resource for the purposes of this study.

### Overdiagnosis and DCIS information

#### Descriptions within websites

UK websites: All but one of the websites included at least some information on overdiagnosis. These nine websites used similar general definitions, referring to it in terms of detection of cancer that would never have caused a problem, harm, symptoms, or become life threatening if left undetected. Three websites used both the terms ‘overdiagnosis’ and ‘overtreatment’ (or terms with this same word root e.g. ‘overdiagnose’), and a further two websites used one of the terms. Six websites mentioned that it was not possible to determine which breast cancers would become invasive, grow quickly, or become life threatening for a given person diagnosed with breast cancer through screening. Seven websites also raised the issue that (most) people diagnosed with breast cancer would receive treatment and that this may be unnecessary in some cases. Furthermore, two websites made a categorical statement regarding the harms of overtreatment in terms of emotional, psychological and physical side effects.

Nine out of the ten websites included some information on DCIS, describing it as a form of cancer that had not invaded the surrounding breast tissue (or spread beyond the milk ducts). Of these nine websites, eight stated that it could become an invasive breast cancer if left untreated and six related the disease to the concept of overdiagnosis. Eight websites also noted that DCIS and screening were closely related, and four made the explicit point that treatment for DCIS was highly effective or that the prognosis was very good. On seven websites, a link to overdiagnosis was made in the form of statements that it was impossible to determine which cases of DCIS would become invasive for a given person.

Australian websites: Six of the eight Australian websites included at least some information on overdiagnosis. Information provided was similar across websites: five described overdiagnosis in terms of detection of breast cancer that would never have caused symptoms, health problems, or become life threatening. Five websites used the term ‘overdiagnosis’ and one also used the term ‘overtreatment’. Six websites stated that it was impossible (or difficult) to determine whether a specific breast cancer would become invasive or harmful. Two websites stated that most people diagnosed with breast cancer would receive treatment, and one stated that unnecessary treatment was associated with harms.

The kinds of information provided about DCIS were similar to the UK websites: four websites gave a description of what it was (e.g. in terms of being a form of breast cancer that is currently confined to the milk ducts and has not become invasive). Two stipulated that DCIS could become invasive if left untreated (or even with treatment), and went on to state that it was not possible to determine whether a particular DCIS case would become invasive and that screening was a common pathway by which women might be diagnosed. Two websites were explicit that DCIS was the type of breast cancer to which overdiagnosis was more likely to apply, and one stated that most women treated for DCIS would not go on to develop invasive breast cancer.

#### Statistics within websites

UK websites: Websites varied considerably in terms of the statistics used to describe the magnitude of overdiagnosis. There were a few recurring examples: the statement that 4,000 women (in the UK) are overdiagnosed or overtreated (through screening) each year was present on three websites. Two websites stated that 3 in 200 women screened (every three years between age 50 and 70 years) would be overdiagnosed or overtreated, and two websites expressed a similar proportion using a different numerator and denominator (17 in 1,000, 129 in 10,000). Two stated a different proportion, seemingly based on a different reference group (women screened over 10 years: 10 in 2,000 or 1 in 200). Six websites stated the ratio of deaths from breast cancer prevented to overdiagnosed cases. In most cases this ratio was explicit, but a small amount of arithmetic was required from the reader for one website. Five websites used estimates derived from the IUKPBCS report when they stated the ratio to be 1:3 or (in one case) 5:17. However, one website stated the ratio to be between 2:1 and 2.5:1 based on research from another group [34]. Furthermore, only three websites referred to the IUKPBCS report explicitly when describing the magnitude of overdiagnosis. The proportion of screen-detected breast cancers that were overdiagnosed or overtreated was stated to be 1 in 4 on one website but 1 in 5 on another. Notably, in seven instances relating to the proportion of women screened who were overdiagnosed, or the ratio of breast cancer deaths prevented to overdiagnosed cases, the statistics provided differed from those provided in the information leaflet for the English NHS Breast Screening Programme. Finally, two websites provided no statistics relating to overdiagnosis.

Australian websites: Despite providing similar conceptual information on overdiagnosis and DCIS, there was relatively little overlap in the kinds of statistical information provided on Australian and UK websites. Two Australian websites referred to World Health Organisation estimates that one or two overdiagnoses of breast cancers occur for each breast cancer death avoided and went on to state that the lifetime risk of overdiagnosis was 3% (although the denominator was not explicitly stated). One other website stated four unique pieces of statistical information, namely i) that previous estimates of the proportion of overdiagnosed breast cancers (out of all breast cancers) in the range of 5–13% had been superseded by more recent studies, ii) that one recent review reported a range of 0–35% (median: 5–9%), iii) that another review reported a range of 1–10%, and iv) the IUKPBCS reported estimates of overdiagnosis in the range of 11–19%. Finally, this website referred to the approximate number of overdiagnoses per 1,000 women screened biennially from age 50 to 74 (around 8; between 2 and 21). This statistic was also presented on one other website. No other statistical information was identified on the remaining four websites.

## Discussion

In this 2015–2016 assessment of the most prominent health websites providing information to the general public on breast cancer screening, we found that almost all UK resources contained information on overdiagnosis and described it using similar terms. In addition, most UK websites explained that overdiagnosis would entail (unnecessary) treatment. Similarly, most UK websites provided information on DCIS, its relevance to breast cancer screening, and either related it to overdiagnosis explicitly or explained that it was unknown whether it would become invasive for a given person. The majority of UK websites also used some form of statistics in order to illustrate the magnitude of overdiagnosis, and it was clear that the IUKPBCS review had been influential. Although it was not always cited explicitly, the statistics most commonly provided (the proportion of women screened who would be overdiagnosed and the ratio of breast cancer deaths prevented to overdiagnosed breast cancers) were derived from the IUKPBCS and there were few exceptions. However, although statistics on overdiagnosis were consistent in many cases, the way that they were presented differed substantially. For example, the probability of overdiagnosis for women undergoing screening was described as a proportion out of 200, 1,000, or 10,000.

A majority of Australian websites also provided information related to overdiagnosis. However, explicit information that a breast cancer diagnosis could lead to unnecessary treatment was less common. Most Australian websites also included information about DCIS although this was more variable: two websites included most of our key information points whereas remaining websites included either minimal information or no information. The Australian website that contained the most information relating to DCIS was also the most detailed website in terms of providing statistical information, including four estimates of the proportion of breast cancers that were overdiagnoses; only three other websites provided statistical details.

This study was strengthened by the selection of websites from the first five pages of Google search results. This was anticipated to generate a wide-ranging list of the most commonly accessed websites, less frequently visited websites, and websites that most users would never see. However, since search engine algorithms adjust results based on variables beyond the specific search terms (e.g. users’ locations), website listings are unlikely to have been exhaustive.

Our findings provide an updated assessment compared to a previous study that reviewed websites in 2002, in which only 26% of websites were found to contain any information on overdiagnosis and overtreatment, and only 37% referred to DCIS [9]. Notwithstanding possible differences in website exclusion criteria between studies, this represents an appreciable change over the past 14 years, most likely reflecting a greater emphasis placed on the issue (and perhaps informed decision-making in general) among those who are professionally involved with screening.

Although online information on overdiagnosis has become more widely available, the relatively limited information about DCIS and statistics on Australian websites may be a concern; to the extent that one considers it important to include this information, it may indicate scope for improvement. However, this finding should be interpreted in the context of the fact that several of the Australian organisations whose websites we included are related to one another, meaning that some websites provided links to more detailed information contained elsewhere.

We also observed variation in how relevant statistical information was presented, especially between UK websites. Given that public understanding of health statistics is limited [35], this may be confusing to readers of multiple websites. In the context of the currently limited understanding of the concept of overdiagnosis among the general public (Ghanouni et al., under review) [3–5], these findings suggest that more could be done to provide clearer and possibly more consistent quantitative information on websites that describe breast cancer screening. This is particularly important where statistics differ from those provided by healthcare providers themselves (e.g. the English NHS Breast Screening Programme and BreastScreen NSW), as these are the set most likely to be encountered by women invited to screening. However, as the most detailed Australian website notes, various estimates of the magnitude of overdiagnosis exist and it is a matter of intense debate as to which are most accurate. Hence, an aim of providing consistent information would need to be balanced against another defensible goal of informing women that there is uncertainty and that several sets of statistics exist.

These findings suggest several possibilities for further research. The analysis consisted primarily of websites that contained content produced by either a health charity or healthcare provider. However, an appreciable amount of public exposure to information on breast cancer screening online is likely to occur on websites without a specific health focus (e.g. social media, news, or video hosting and streaming services) [36]. It may be useful for future research to explore the nature of overdiagnosis information on websites such as Twitter, YouTube, and those of mainstream media outlets. Furthermore, in order to limit the role of subjective personal opinions in our evaluations, we limited our information coding to broad descriptive categories. Future studies could also examine other aspects relating to how that content is communicated, such as information placement (e.g. if information on overdiagnosis was less discoverable due to being located at the bottom of a lengthy page), tone (e.g. whether information was empathetic or emotionally detached), and the use of visual elements to describe statistics (e.g. graphs or icon arrays).

In conclusion, we found that a higher proportion of UK and Australian health websites explained overdiagnosis and DCIS than websites accessed in 2002, although there was scope for improvement within both sets. Descriptive information on the concepts was relatively consistent, although information relating to DCIS and the relevant statistics were less prevalent among Australian websites. Statistical information varied considerably across UK websites, potentially causing confusion. Future work could extend the area of study to websites that are not health-specific and describe more subtle characteristics of website information.
